# Essential Function of Dynamin in the Invasive Properties and Actin Architecture of v-Src Induced Podosomes/Invadosomes

**DOI:** 10.1371/journal.pone.0077956

**Published:** 2013-12-09

**Authors:** Olivier Destaing, Shawn M. Ferguson, Alexei Grichine, Christiane Oddou, Pietro De Camilli, Corinne Albiges-Rizo, Roland Baron

**Affiliations:** 1 Institut Albert Bonniot, Université Joseph Fourier; Université Joseph Fourier site Santé, Grenoble cedex, France; 2 Department of Cell Biology, Howard Hughes Medical Institute, Program in Cellular Neuroscience, Neurodegeneration and Repair, Yale University School of Medicine, New Haven, Connecticut, United States of America; 3 Department of Medicine, Harvard Medical School, Endocrine Unit, Massachusetts General Hospital, Boston, Massachusetts, United States of America; 4 Department of Oral Medicine, Infection and Immunity, Harvard School of Dental Medicine, Boston, Massachusetts, United States of America; West Virginia University, United States of America

## Abstract

The large GTPase dynamin plays a key role in endocytosis but is also localized at numerous actin rich sites. We investigated dynamin functions at podosomes/invadosomes, actin-based cellular adhesion structures implicated in tissue invasion. Podosomes/invadosomes are constituted of long F-actin bundles perpendicular to the substratum (actin cores), connected to randomly arranged F-actin fibers parallel to the substratum (actin cloud). We show here that dynamin depletion in v-Src-transformed fibroblasts triggers a massive disorganization of podosomes/invadosomes (isolated or in rosettes), with a corresponding inhibition of their invasive properties. The action of dynamin at podosomes/invadosomes requires a functional full-length protein, suggesting that the effects of dynamin at these sites and in membrane remodelling during endocytosis are mediated by similar mechanisms. In order to determine direct effect of dynamin depletion on invadosome, an optogenetic approach based on the photosensitizer KillerRed was developed. Acute dynamin photo-inactivation leads to a very rapid disorganization of invadosome without affecting focal adhesions. Dynamin therefore is a key regulator of the architecture of actin in podosomes/invadosomes.

## Introduction

The large GTPase dynamin, a member of a family of GTPases implicated in membrane remodeling, plays a critical role in membrane fission during clathrin mediated endocytosis [1], [2], [3]. When this endocytic reaction also involves actin, the spatial and temporal recruitment of dynamin closely correlates with the recruitment of actin and actin regulatory proteins, such as cortactin and components of the N-WASP and Arp2/3 actin-nucleating complex [4]. In addition, dynamin is associated with numerous other actin-dependent structures, primarily those nucleated by N-WASP, cortactin and the Arp2/3 complex. These include: lamellipodia, phagocytic cups, immunological and neuronal synapses, actin comets, circular dorsal ruffles, podosomes and invadopodia [5], [6], [7], [8], [9], [10]. Moreover, pharmacological agents that disrupt dynamin function impair actin dynamics at lamellipodia and affect cell spreading [11]. In agreement with these findings, a dominant negative mutant of dynamin, dynamin K44A, which blocks clathrin-mediated endocytosis, also reduces the formation of several actin-rich structures including dorsal circular ruffles [10], [12], podosomes [13], [14] and invadopodia [15]. Similarly, depletion of dynamin 2 by siRNA treatment leads to abnormal organization and distribution of podosomes in osteoclasts, but still without entirely preventing their formation [14], [16].

A mechanistic link between the function of dynamin and the actin cytoskeleton is supported by both direct and indirect interactions. The C-terminal proline-rich domain (PRD) of dynamin binds a variety of actin regulatory proteins, such as cortactin, Tuba, intersectin and members of the Toca family [17], [18], [19], [20]. Purified dynamin 2, in the presence of cortactin, binds, bundles and remodels actin fibers upon GTP hydrolysis, leading to fraying of the F-actin bundles [21]. Additionally, dynamin was reported to modulate elongation of short F-actin filaments by competing with the capping protein gelsolin [22].

However, studies of fibroblasts that lack dynamin, *i.e.* dynamin 1 and 2 double KO (DKO) fibroblasts, revealed defects in the organization of circular dorsal ruffles induced by growth factors (Hongying Shen, Shawn Ferguson and Pietro De Camilli, unpublished observations), but failed to reveal prominent structural changes in several actin-dependent structures such as focal adhesions, lamellipodia and stress fibers [1], [23]. The major actin-related effect observed in a first analysis of these cells was a prominent accumulation of actin and actin-nucleating proteins at the necks of arrested endocytic clathrin coated buds [1], [23]. These findings supported a close link between actin and the endocytic function of dynamin and also revealed that at least at these sites the recruitment and polymerization of actin is not dynamin-dependent. However, the role of dynamin in podosomes or invadopodia remains elusive.

Podosomes and invadopodia are adhesive mechanosensory modules composed of a dense F-actin core surrounded by a ring of adhesion molecules (Albiges-rizo et al., 2009). The distinction between podosomes and invadopodia is still a matter of debate. Since the exact relationship between invadosomes, podosomes and invadopodia is unclear, degradative F-actin-rich structures induced by v-Src will be grouped here under the term invadosomes [24], [25], [26]. In this report, we have investigated the function of dynamin in invadosomes, focusing in particular on the so-called rosettes, small ring-shaped adhesion structures that result from the spontaneous self-assembly of individual invadosomes and their surrounding F-actin cloud. Such structures offer interesting advantages as a model to study the role of dynamin in actin dynamics. They are very prominent, and thus easily visualized. Their 3D ultrastructure is well characterized and composed of long columns of tightly bundled F-actin filaments perpendicular to the substratum (actin cores representing individual invadosomes), which are connected by an actin cloud composed of radial F-actin filaments parallel to the substratum [27], [28]. Rosettes are highly dynamic structures, which form, expand in diameter, often fuse with each other and disappear due to continuous remodeling resulting from the coordinated assembly of new invadosomes at their outer rim and disassembly of older ones at the inner rim [29], [30], [31]. In osteoclasts their fusion and expansion leads to the formation of a characteristic podosome peripheral belt and sealing zone on bone [29], [30], [32], [33].

To study the role of dynamin at invadosomes, we have explored the formation of rosettes in dynamin 1 and 2 double knockout mouse embryonic fibroblasts (MEF) by the expression of a constitutively active mutant of the tyrosine kinase Src, v-Src, as previously described [13], [34], [35]. Our results demonstrate that the formation of invadosome rosettes is blocked in the absence of dynamin and that formation of invadosomes requires both a functional GTPase module and the assembly properties of dynamin. Furthermore, minute-scale loss-of-function of dynamin achieved by photo-inactivation rapidly disrupted invadosome rosettes, while leaving the formation of other structures such as focal adhesions, lamellipodia, or stress fibers unaffected. Thus, dynamin plays an important role in defining the architecture and dynamics of the actin cytoskeleton at invadosomes.

## Results

### Dynamin and actin in the dynamics of invadosomes in MEF-v-Src cells

It was previously shown that a dominant negative mutant of dynamin 2 (Dynamin K44A) impairs invadosome formation [13], [15]. However, dominant negative interference functions, at least in part, by sequestering binding partners of the mutant proteins. Determination of the function of a protein additionally requires studies of the effect of its deletion. To identify the role of dynamin in these structures, we analyzed invadosomes in cells genetically depleted of dynamin. Mammalian genomes contain 3 dynamin genes that encode dynamin 1, 2 and 3, respectively. Dynamin 2 is ubiquitously expressed and represents the major isoform expressed in most non-neuronal cells, while dynamin1 and 3 are expressed primarily in the nervous tissue [36], [37]. To generate dynamins-depleted cells in our experiments, we used MEFs derived from Dnm1^−/−^; Dnm2^LoxP/LoxP^ mouse embryos [1]. Thus, these cells only lacked dynamin-1, *i.e.* single KO (SKO MEFs, Dnm1^−/−^, Dnm2^LoxP/LoxP^) and were used to generate a dynamin1/dynamin 2 double KO genotype upon Cre recombinase expression (DKO MEFs ie Dnm1^−/−^;Dnm2^−/−^). Invadosome rosettes were induced in these cells by expressing v-Src ([13], [35], [38], the constitutively active form of the proto-oncogene c-Src in order to obtain SKO-v-Src-MEFs and DKO-v-Src-MEFs (Dnm1^−/−^;Dnm2^−/−^ [13], [35], [38].

**Figure 1:**
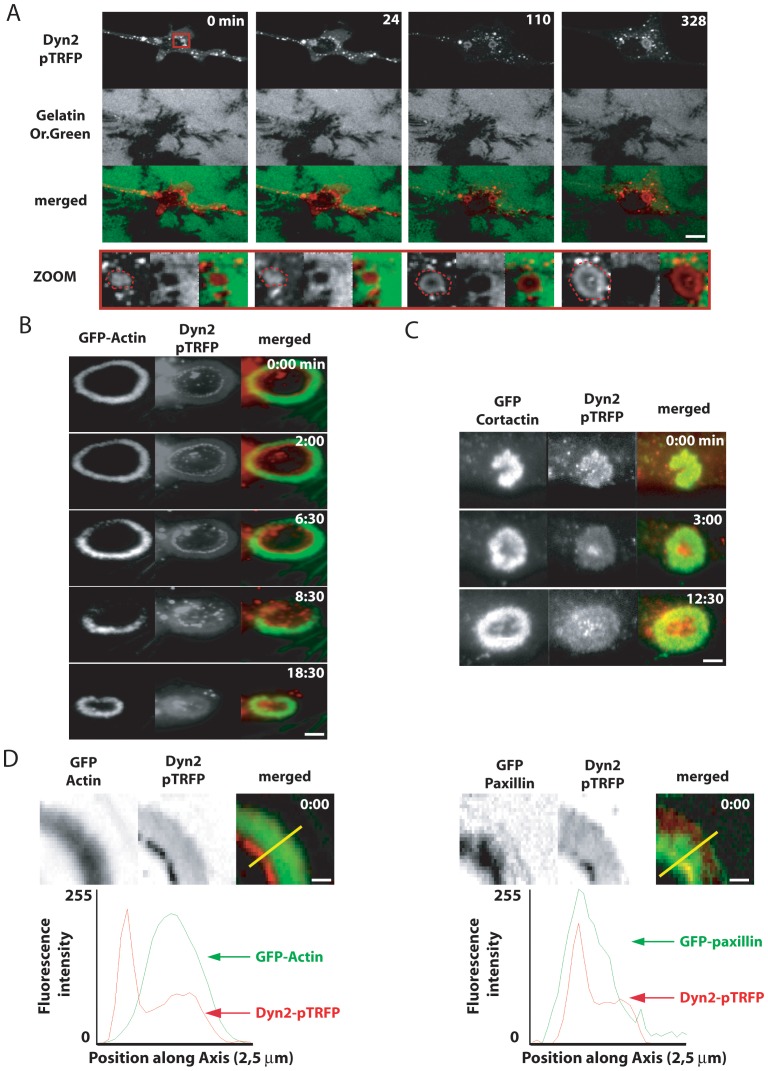
Dynamin is dynamically associated with actin reorganization and extracellular matrix degradation activity of invadosome rosettes. A) Extracted images from time serie (min) from representative observations of SKO-v-Src-MEFs expressing Dyn2-pTRFP and spread on gelatin-OregonGreen degradable surface. As shown in the zoom corresponding to the red square, dyn2-pTRFP is localized in rosette (red dash circle), and is present all along the degradative activity of the structure. B–C) Extracted images from time serie (min∶s) from representative observations of SKO-v-Src-MEFs expressing Dyn2-pTRFP in association with GFP-actin and GFP-cortactin. The dynamic of invadosome ring is based on a treadmilling movememnt based on the polymerization of new actin structures at the outer rim and depolymerization of older actin structures at the inner rim of the ring. Dyn2-pTRFP is perfectly colocalized with GFP-actin and GFP-cortactin during expansion of the invadosome ring. D) Zoom on invadosome ring expansion. Quantification of fluorescence intensity (8bits color image coded from 0 to 255 levels) of Dyn2-pTRFP and GFP-actin or GFP-paxillin allowed generating the intensity profile intensity along the yellow line (24 px) in the merged image. Dyn2-pTRFP colocalized either with GFP-actin either with GFP-paxillin present also at the region of actin depolymerization (inner rim). Scale bar = 5 µm (A), 3 µm (B, C) and 0,5 µm (D).

As previously observed for the localization of endogenous and transfected dynamin in v-Src-expressing wild type fibroblasts [13], [15], Dyn2-pTRFP expressed in v-Src-Dnm1^−/−^,Dnm2^LoxP/LoxP^ cells accumulated in numerous spots. Some of these spots formed a ring structure (Fig. 1A, red dashed circle in the zoom) that colocalized with actin (Fig. 1B). Furthermore, when MEFs were grown on a gelatin-Or.Green-containing substrate, these rings colocalized with areas of digested gelatin and their expansion occurred concomitantly with the increase of the digested area (Fig. 1A). Thus these rings have the typical characteristics of invadosomes (Fig. 1A, movie S1).

**Figure 2:**
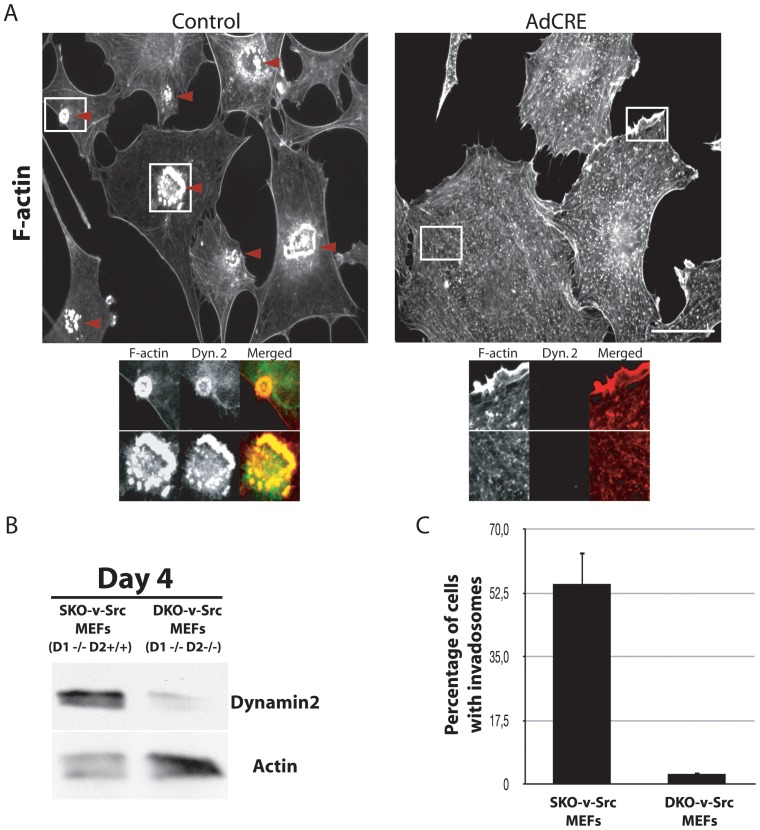
Dynamin is essential for invadosome formation. A) DKO-v-Src-MEFs cells were obtained 7 days after CRE recombinase expression. Dynamin depletion induced the desorganization of invadosomes (isolated or organized into rings) visualized by phalloidin staining. Endogenous dynamin 2 is concentrated in invadosome and this staining is specific while not present in DKO-v-Src-MEFs cells. Moreover, higher magnification of the areas in the white squares is showing the replacement of invadopodia in DKO-v-Src-MEFs by small cytosolic actin spots. B) Endogenous dynamin 2 is almost not detectable in cells 4 days after expression of the CRE recombinase. C) Quantification of the percentage of cells forming invadosome revealed that almost 95% of SKO-v-Src-MEFs treated with the CRE recombinase are not forming these structures 7 days post infection (n = 650 counted cells per conditions). Scale bar = 10 µm (A).

Rosette expansion involves assembly of new invadosomes at the outer edge of the ring and depolymerization at its inner edge [30], [35]. Dyn2-pTRFP (Fig. 1A), like endogenous dynamin 2 (Fig. 2A), was observed throughout the ring in Dnm1^−/−^; Dnm2^LoxP/LoxP^ cells, but with a sharp accumulation at its inner edge (Fig. 1B). This intensity profile was similar to that of paxillin (paxillin-GFP) (Fig. 1D), a protein known to be involved in invadosome disassembly (Fig. 1D) [30]. The diffuse pool of dynamin colocalized with GFP-actin and GFP-cortactin throughout the ring (Fig. S1B, C, D and movie S2, S9 and S10). Thus, dynamin is associated with dynamic actin networks in invadosomes, particularly at the inner rim of rosettes, an area where disassembly of these structures is taking place.

### Dynamin is essential for invadosome formation

Suppression of dynamin expression in SKO-v-Src-MEFs, was achieved by expression of Cre-recombinase through adenoviral transduction. This procedure resulted in a cell population that at 4 days post-transduction had dramatically lower levels of dynamin 2 expression [the residual dynamin 2 most likely reflects cells where recombination did not occur [1] and no detectable expression of dynamin 1. In these DKO-v-Src-MEFs cells, both isolated invadosomes and invadosome rosettes were almost absent (Fig. 2A, C; movie S3). In contrast, cells that only lacked dynamin 2, SKO-v-Src-MEFs formed invadosomes (data not shown) indicating that the endogenous expression of dynamin 1 expression is sufficient to compensate the functions of dynamin 2, as shown recently for clathrin mediated endocytosis [1]. These results show an essential function of dynamin in the formation and maintenance of these characteristic actin-based structures.

Based on these results, all subsequent experiments aimed at exploring the function of dynamin in invadosomes were carried out exclusively in DKO-v-Src-MEFs. Importantly, even though the DKO-v-Src-MEFs cells did not form invadosomes, they still generated other actin based structures such as stress fibers, lamellipodia, ruffles, and the previously described actin spots at arrested endocytic clathrin coated pits [1], which are clearly evident in Fig. S1B.

### Dynamin is essential for invasion

**Figure 3:**
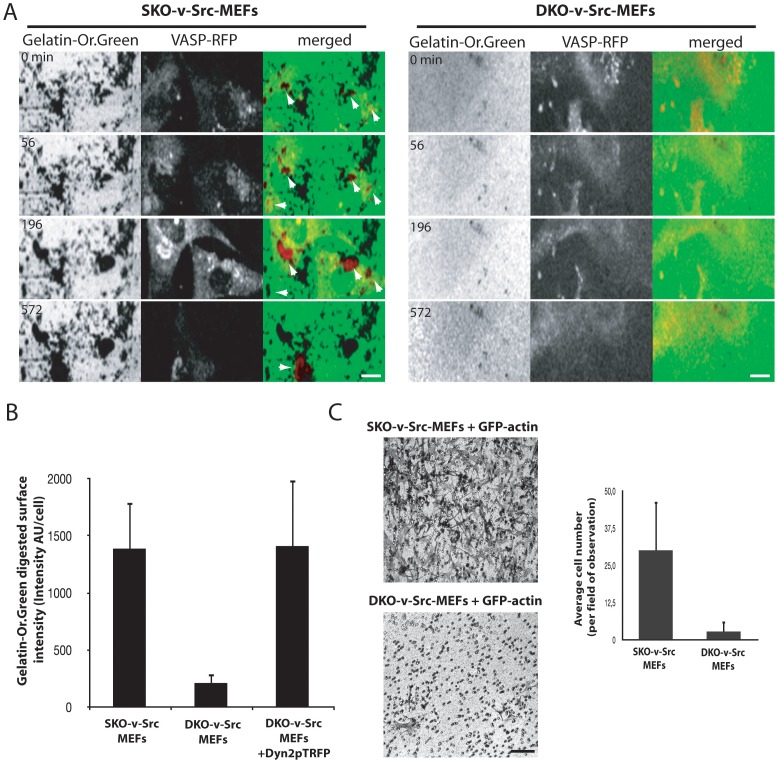
Desorganization of invadosome induced by dynamin depletion inhibits degradative and invasive properties of DKO-v-Src-MEFs. A) Extracted images from time serie from representative observations of SKO-v-Src-MEFs and DKO-v-Src-MEFs expressing VASP-RFP and spread on Gelatin-OregonGreen degradable surface. VASP-RFP is localized in adhesion structures as invadosome rings and abolished degradative properties of DKO-v-Src-MEFs (B). C) Invasion is monitored by quantification of the average number of cells per field of observation (32×, 20 fields measured per conditions) of SKO-v-Src-MEFs and DKO-v-Src-MEFs migrating throug a thick layer of matrigel recovering a Boyden chamber in response to a serum chemiotactic gradient. Dynamin depletion inhibits invasive properties of DKO-v-Src-MEFs. Scale bar = 10 µm (A), 50 µm (B).

We next sought to determine whether the loss of invadosomes upon dynamin deletion is associated with a defect in the ability of cells to degrade extracellular matrix. To test this, DKO-v-Src-MEFs expressing VASP-RFP to visualize invadosomes, were grown on a digestible layer of gelatin-Or.Green. Live cell imaging revealed that only SKO-v-Src-MEFs cells digested the extracellular matrix at invadosome contacts (indicated by white arrow), whereas DKO-v-Src-MEFs cells did not (Fig. 3A, B), despite the presence of constitutively active Src in these cells. Quantification of the gelatin digested per cell confirmed the importance of dynamin in the ECM degradative capacity of these cells (Fig. 3C).

To determine the importance of dynamin in the general context of invasion, the invasive properties of SKO-v-Src-MEFs and DKO-v-Src-MEFs cells were analyzed. For that purpose, SKO-v-Src-MEFs and DKO-v-Src-MEFs expressing either GFP-actin (as a control of infection) were harvested, seeded above a matrigel layer on the top of a Boyden chamber and allowed to migrate towards the bottom of the transwell containing a chemoattractant for 20 h. Next, cells that did not invade the matrigel layer were removed and cells that passed through the filter of the Boyden chamber were fixed and stained with crystal violet. In agreement with a defect in extracellular matrix degradation, dynamin depletion inhibited the invasive properties of DKO-v-Src-MEFs through the thick layer of matrigel (Fig. 3C). Note that even though the migration of DKO-v-Src-MEFs cells in matrigel was largely reduced, their ability to form lamellipodia and to move was not entirely blocked but simply reduced by less than 50%, as shown by a monolayer wounding test (Fig.S2). Thus, the defective invadosome formation observed in DKO-v-Src-MEFs is associated with an inhibition of their invasive properties.

### Invadosomes are dependent on dynamin's catalytic and assembly activities

**Figure 4:**
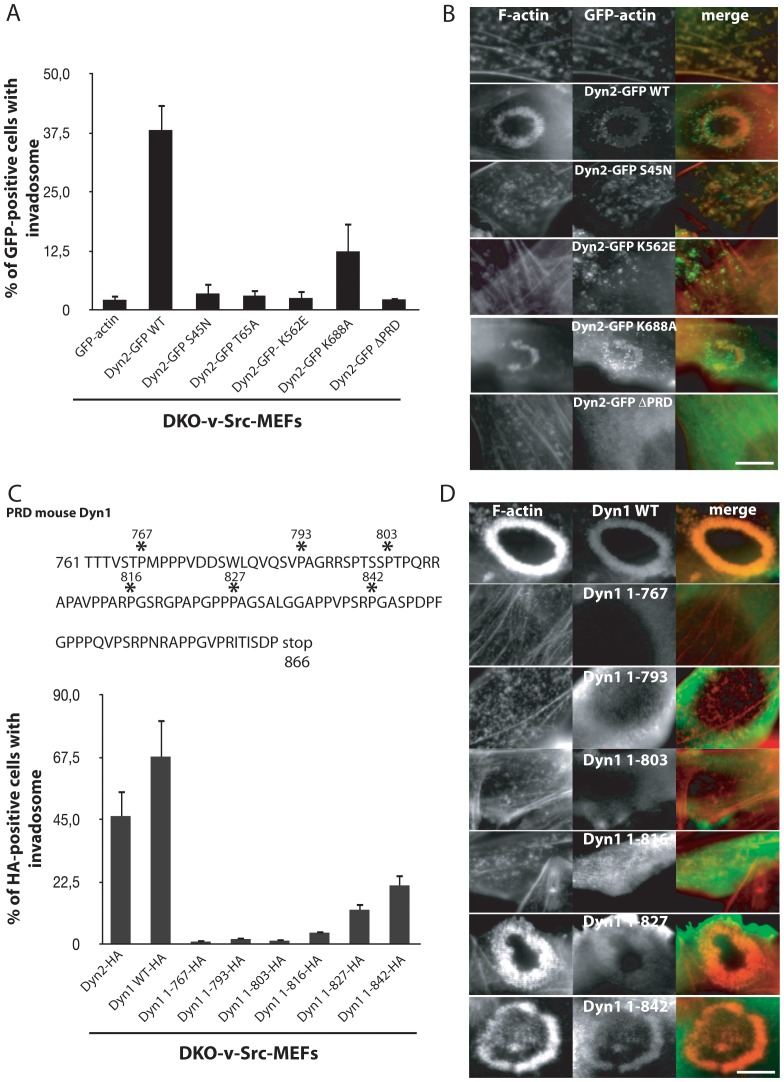
Dynamin function in invadosome is dependent of a coordinated activity of its GTPase, PH and GED domains. A) Re-expression of multiple dynamin mutants (450≤n<650 invadosomes) revealed that GTPase, PH domain and PRD domains have cooperative functions to rescue invadosome formation in in DKO-v-Src-MEFs. B) The PRD is only essential domain for dynamin localization in F-actin structures (small actin punctates or invadosomes). C) Amino-acyl sequence of the PRD domain of mouse dynamin 1 where the sites of the multiple stop in the PRD sequence are indicated by an asterisk (*). Increasing PRD length is essential to rescue invadosome formation in DKO-v-Src-MEFs (450≤n<650 invadosomes). D) The ability to rescue invadosome formation is linked with the ability to relocalize to F-actin structures. Scale bar = 5 µm (A, B).

It was of interest to determine whether the same properties that are key to the endocytic function of dynamin are also essential for its role in invadosome formation and dynamics. To address this question, we performed rescue experiments by expressing wild type or mutant dynamin 1 or 2 fused to an HA epitope tag or GFP in DKO-v-Src-MEFs and determined the percentage of GFP-expressing cells with invadosomes. Expression of GFP-actin in DKO-v-Src-MEFs was used as a control and did not restore the formation of invadosomes. Expression of either dyn2-GFP (Fig. 4A.), HA-dyn2 or HA-dyn1 (Fig. 4C) restored the formation of invadosomes in DKO-v-Src-MEFs cells (Fig. 4), while mutant dynamins harbouring mutations that affect its GTPase cycle by decreasing the affinity for GTP - dyn2-GFP S45N and T65A mutants [39] – did not (Fig. 4A, B). The K688A mutation in dynamin 2, which in human dynamin 1 (L694A) greatly impairs self-assembly *in vitro* and *in vivo* [39], [40], [41], [42], produced only a minimal rescue of invadosome formation (Fig. 4A) No rescue was produced by the pleckstrin homology (PH) domain K562E mutant of dynamin 2 that corresponds to a centronuclear myopathy patient mutation [43]. Importantly, all these mutants were still recruited to actin spots (Fig. 4B), that represent in part arrested clathrin coated pits (Fig. S1A,B), speaking against a defect due to inefficient targeting to the actin cytoskeleton. In contrast, dyn2-GFP and dyn1-HA deleted of their C-terminal Pro-Rich Domains (PRD region) neither restored invadosomes, nor were localized to cytosolic actin spots, demonstrating the importance of this region, which contains binding sites for cortactin and actin regulatory proteins, for dynamins targeting [21], [44]. Beside there is no significant difference between dyn2-GFP and dyn2-HA to rescue invadosome formation, it is interesting to notice that a statistical difference was seen between dyn2-HA and Dyn1-HA in rescue experiments. Analysis of a series of dynamin 1 constructs with progressive C-terminal truncations demonstrated that the region following amino acid 816 of the PRD is critical for the restoration of invadosomes in DKO-v-Src-MEFs (Fig. 4C). We conclude that, as in the case of the function of dynamin in endocytosis, the coordinated actions of all main domains of dynamin are needed for its role in invadosome formation.

### Invadosome disruption by photoinactivation of dynamin

The striking effect of the lack of dynamin on the formation of invadosomes could be due to long-term effects and adaptive changes in signalling pathways. However, the analysis of phosphoproteins that reflect the activation state of the main signalling pathways that regulate invadosomes (cortactin and Src activation) did not reveal major differences (except for an increase of Erk activity) between SKO-v-Src-MEFs MEFs, DKO-v-Src-MEFs and DKO-v-Src-MEFs re-expressing Dyn2-GFP (Fig. S3A). The use of Erk inhibitor did not rescue invadosome formation in DKO-v-Src-MEFs, suggesting that the increase of this signalling pathway is not responsible for the observed phenotype (Fig. S3B). To further investigate the role of dynamin in invadosome formation, and to complement experiments in cells that chronically lack dynamin, we developed a strategy to disrupt dynamin function at the minute time-scale by using photoinactivation.

**Figure 5:**
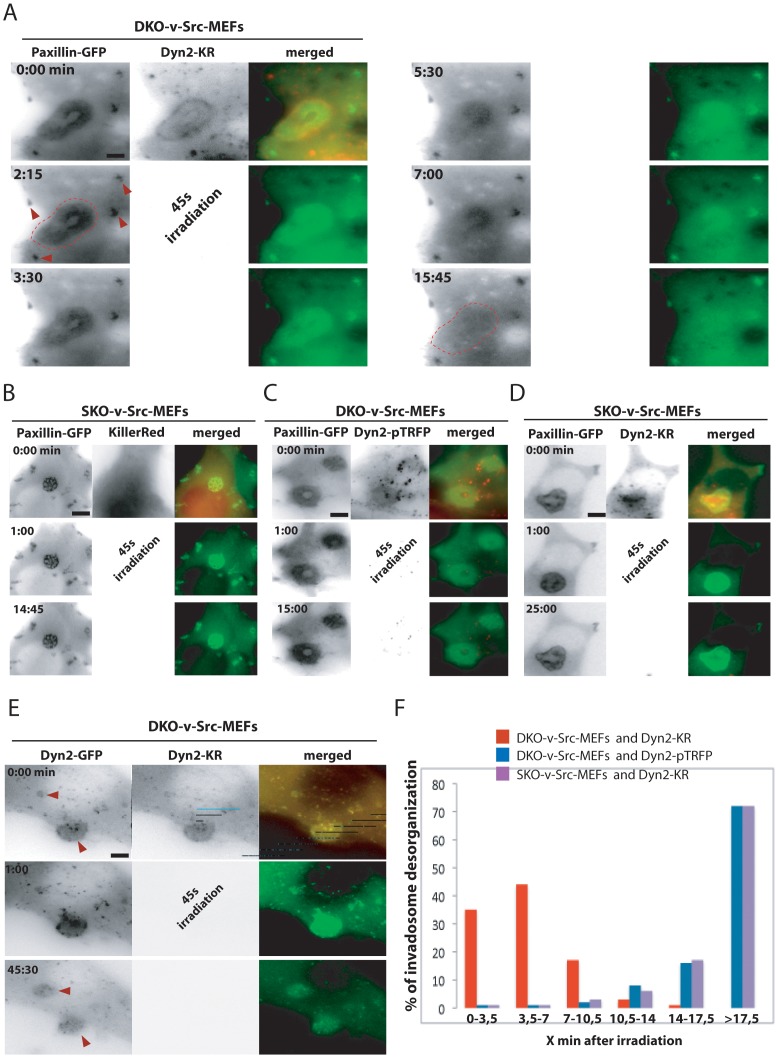
Specific dynamin photoinactivation led to the rapid invadosome disorganization. A) Extracted images from time serie (min∶s) from representative observations of DKO-v-Src-MEFs expressing dyn2-KR and GFP-paxillin. Dyn2-KR is functionnal while localized properly and rescued invadosome formation. KillerRed 45 s light irradiation is followed by the rapid dissociation of invadosome ring (dashed line) stained by GFP-paxillin also localized in focal adhesions (red arrows). B) Extracted images from time serie (min∶s) from representative observations of SKO-v-Src-MEFs expressing KillerRed (KR) alone and GFP-paxillin. Non-localized ROS production after light irradiation of KillerRed alone does not destabilize adhesions structures. C) Extracted images from time serie (min∶s) from representative observations of DKO-v-Src-MEFs expressing dyn2-pTRFP (same excitation/emission spectrum but much more photostable than KillerRed) and GFP-paxillin. Light irradiation without ROS production is not sufficient to dissociate invadosome structures. D) Extracted images from time serie (min∶s) from representative observations of SKO-v-Src-MEFs expressing GFP-paxillin and dyn2-KR. In presence of endogenous dynamin, photoinactivation of dyn2-KR has no effect on invadosome organization showing the localized and specificity of ROS produced by the photosensitizer on the proteins fused to it. E) Extracted images from time serie (min∶s) from representative observations of DKO-v-Src-MEFs expressing dyn2-GFP and dyn2-KR. ROS production at the level of the GTPase has no effect on dyn2-GFP stability visualizing and confirming the previous experiment. F) Distribution of the percentage of cells where invadosome structures is disorganized×min after light irradiation. The >17,5 min category corresponds to experiments where invadosome presented a life-span superior to 17,5 min (distributed between 20 and 40 min, depending of the duration of each movies) and pulled altogether. 16 to 56 cells per conditions were monitored. Scale bar = 2 µm (A, E), 4 µm (B, C, D).

To this end, the GFP moiety of dyn2-GFP was replaced by the photosensitizer Killer Red™ (KR) protein which is able to locally produce reactive oxygen species (most likely singlet oxygen, Roy et al., 2010) in response to red light irradiation, and thus to inactivate the protein fused to KR [35], [45]. Dyn2-KR was expressed in DKO-v-Src-MEFs along with paxillin-GFP, a marker of adhesion structures. The ability of Dyn2-KR to functionally replace WT dynamin was confirmed by its localization at invadosomes and by its ability to rescue invadosome formation in DKO-v-Src-MEFs (Fig. 5A).

After 45 s irradiation with red light, invadosomes became disorganized (dashed line) without any change in focal adhesions (red arrows, Fig. 5A and movie S4). After 10 minutes cells resembled DKO-v-Src-MEFs cells due to the absence of invadosomes (Fig. 5A,F). That local OS production disorganizes invadosome structure specifically through inactivation of dyn2-KR was confirmed by the observation that a 45 s irradiation of SKO-v-Src-MEFs (i.e. cells that express endogenous dyn2) also expressing GFP-paxillin and large amounts of free KillerRed did not have any effect on invadosomes (Fig. 5B, Fig. S4 and movie S5). Toxicity of the 45 s light irradiation itself was tested by replacing KR with pTRFP, a more photostable fluorescent protein with an identical excitation/emission spectrum as KR, which did not induce invadosome disorganization after irradiation, (Fig. 5C, F and movie S6). Moreover, the KillerRed strategy had no effect on endogenous proteins, more precisely on non-tagged dynamin as irradiation of SKO-v-Src-MEFs expressing dyn2-KR did not induce invadosome disorganization (Fig. 5D, F and movie S7). Confirming this result, photoinactivation of dyn2-KR in DKO-v-Src MEFs expressing dyn2-GFP and dyn2-KR had no effect on invadosomes and on the localization dyn2-GFP at invadosomes (red arrow, Fig. 5E, Fig. S4 and movie S8).

**Figure 6:**
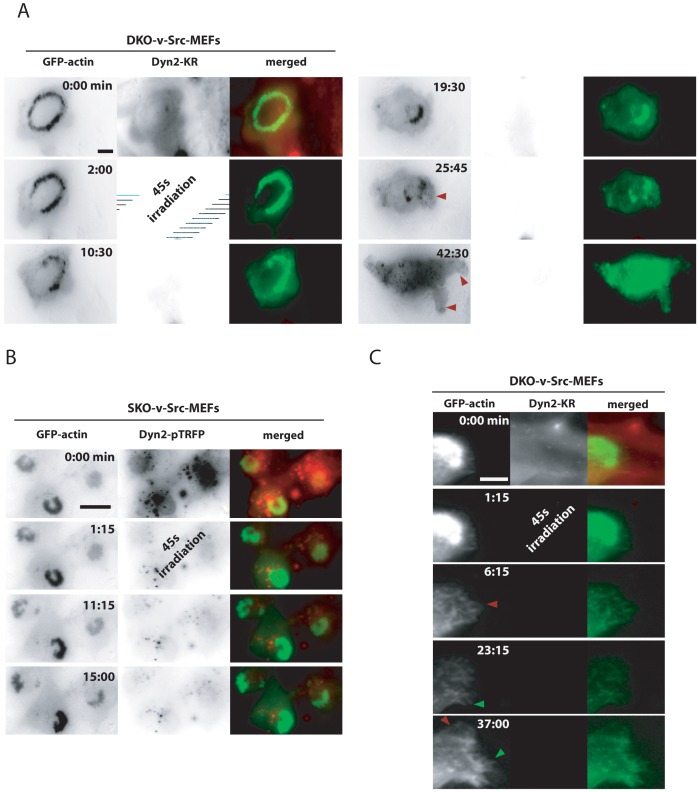
Photoinactivation revealed a direct and specific function of dynamin in actin organization of invadosome. A–B) Extracted images from time serie (min∶s) from representative observations of DKO-v-Src-MEFs expressing dyn2-KR and GFP-actin. Dynamin photoinactivation leads to a rapid desorganization of actin in invadosome ring and the slow formation of lamellipodia (red arrows) and accumulation of cytosolic actin spots. B) Filopodia (green arrow) and lamellipodia (red arrow) are formed independently of dynamin. Scale bar = 2 µm (A), 4 µm (B, C).

The consequences of acute dynamin inactivation on the actin cytoskeleton were further assessed in DKO-v-Src-MEFs expressing both GFP-actin and dyn2-KR. While the invadosome ring, as detected by the GFP signal, was disrupted a few minutes after dynamin photo-inactivation (Fig. 6A), the photo-inactivated cell was still able to spread, to develop lamellipodia (red arrows, Fig. 6A, C) and to accumulate actin spots, suggesting no drastic interference with the actin cytoskeleton. As shown above, photoinactivation of dyn2-pTRFP, which is expected to produce less reactive oxygen species, has no effect on invadosomes (Fig. 6B). The rapid disruption of invadosomes upon dynamin depletion (few minutes) contrasts with the subsequent formation of lamellipodia (after 15 min) confirming the direct and specific function of dynamin on actin structures at invadosomes. In a separate experiment, dyn2-KR inactivation did not block the formation of filopodia either (green arrow, Fig. 6C) further showing that the organization and architecture of these structures are also independent of dynamin. Thus, dynamin is essential for the minute-to-minute maintenance of invadosomes.

## Discussion

Invadosomes and invadopodia, collectively now called invadosomes [24], [25], [26] are actin-driven structures that coordinate attachment to the extracellular matrix (ECM) with its degradation [24], [31], [46]. We show here that invadosomes formation is impaired in cells that lack dynamin. Furthermore, acute disruption of dynamin function by photo-inactivation led to the rapid disappearance of pre-existing invadosomes. Thus, the GTPase dynamin plays a critical importance both in the formation and maintenance of invadosomes.

Based i) on the presence of dynamin at such structures, ii) on the frequent occurrence of tubular plasma membrane invaginations within invadosome/invadosome rosette and iii) on the property of dynamin to tubulate membranes, it had originally been proposed that dynamin was responsible for the tubular invaginations, possibly linking them to their actin shell [13]. Supporting this interpretation was the similar diameter of these tubules to the diameters of tubules generated by purified dynamin in the presence of lipid bilayers in vitro [47], [48]. However, it was subsequently found that dynamin is not restricted to the membrane interface at invadosomes but that is localized throughout the actin core and the surrounding actin cloud [14], [49]. Furthermore, tubules such as those found in invadosomes can be generated by several other proteins [50], [51], [52], including dynamin binding proteins concentrated at invadosomes [53], [54], [55], [56].

Dynamin is now known to interact directly or indirectly with a variety of actin regulatory proteins thus explaining its presence in and around invadosomes [13], [14], [15], as well as in a variety of actin-rich structures, primarily those nucleated by the Arp2/3 complex and WASP/Wave family proteins [8], [9], [10], [18], [57]. Interestingly, several of the numerous proteins that bind the proline-rich C-terminal region of dynamin also bind proteins that have a role in actin nucleation. Examples include, but are not limited to, cortactin, Tuba and proteins of the FBP17/Toca/CIP4 family [19], [20]. Some of these proteins were first identified for their function as actin regulatory factors [58], [59] and only subsequently were found to bind dynamin and to have a potential role in endocytosis. Others were first identified as endocytic factors and later reported to have a role in actin regulation [11], [50], [60]. A direct interaction of dynamin with actin has also been recently reported [22]. While our findings add new evidence for a close link between dynamin and actin and also conclusively establish the importance of dynamin for invadosome/invadosome function, the mechanism(s) that mediate dynamin's action at these sites remain an open question.

One possibility is that the importance of dynamin for invadosome dynamics may be related to its endocytic role, in spite of lack of clear evidence for intense endocytic activity occurring at invadosomes. For example, since actin has been implicated in several forms of endocytosis, the binding of dynamin to actin and actin regulators may help to concentrate dynamin at endocytic sites and thus to generate the focal concentration of dynamin that is required for its polymerization once the neck of an endocytic pit is generated. In this scenario, which is supported by the concept that membrane remodeling is the key function shared by all members of the dynamin superfamily [61], [62], [63], the interaction of dynamin with actin would simply play an ancillary role to its endocytic function. However, dynamin depletion led to a general disassembly of actin at invadosome sites whereas the absence of this GTPase is associated with an intense actin polymerization at arrested clathrin-coated pits [1]. This speaks against the possibility that dynamin inactivation may produce the arrest of endocytic intermediates at invadosomes. Furthermore, if the importance of dynamin in invadosome dynamics was simply an indirect consequence of its function in endocytosis, one would expect a delay between dynamin inactivation and disappearance of the invadosome. While conditional Cre-recombinase-dependent KO experiments, which require days for the disappearance of the protein, are compatible with such a delay, the dramatic and very rapid (tens of seconds/minutes) effects of the acute photo-inactivation of dynamin speaks against this possibility and favor a direct role of dynamin in the 3D architecture of the actin meshwork at invadosomes.

A related possibility is that the role of dynamin in invadosome dynamics may reflect the effect that endocytosis of receptors has on their signaling output. Lack of dynamin may disrupt normal signaling by preventing the internalization of receptors (including integrins) with an impact on signaling that may occur both at the cell surface and on endosomes. While our results do not provide evidence for this mechanism, this remains a plausible scenario that may contribute to other dynamin functions at invadosomes.

Yet another possibility is that dynamin may have independent actions in endocytosis and in actin dynamics. Its function at invadosomes may be independent of its endocytic function since, based on current knowledge, invadosomes do not appear to have a predominant endocytic role. One could speculate that the proline-rich domain of dynamin, i.e. the portion of the protein that links it to most actin regulatory proteins, may have a function at least partially independent from the portions of the protein involved in endocytosis. However, our experiments with mutant dynamin show that all dynamin domains, including the GTPase domain and it catalytic activity, are needed for the action of dynamin at invadosomes. Recent crystallographic and cryo-EM studies [64], [65], [66], [67] have shown how the functions of the different domains of dynamin are interdependent on each other. Such studies have shown that the polymeric helical organization of dynamin around a tubular template and the interaction of different rings of the spiral with each other are needed to promote the GTPase activity that is responsible for dynamin's action. While such a helical organization has clear relevance for the membrane fission activity of dynamin at the tubular neck of an endocytic pit, its significance in other contexts, such as in the regulation of actin function, remains unclear. Wild type dynamin can oligomerize into a spiral around microtubules [68] and actin bundles [21] in vitro, but it remains to be seen whether this property has a function in living cells. No transient hot spots of dynamin that may correspond to transient formation of ring-like structures, similar to the hots spot of dynamin visible at endocytic sites, can be seen in the actin clouds that surround invadosome cores.

Based on all these considerations, a direct role of dynamin in the regulation of actin at invadosomes remains plausible. However, the precise mechanism through which dynamin may achieve its effects at these structures remains a most interesting priority for future studies. Elucidating such mechanisms will have important implications because the recent developments of drugs that block dynamin activity opens the possibility of targeting its function in pathological conditions where invadosomes/invadopodia are implicated [11], [69].

## Material and Methods

### Antibodies and reagents

Antibodies for immunoblotting and immunofluorescence were obtained from the following commercial sources: mouse anti-GFP (clone b2, Santa Cruz), rabbit anti-PhosphoTyr 418 Src (Invitrogen, Carlsbad, CA), mouse anti-Src (GD11, Millipore, Billerica, MA), anti-Phospho Tyr 421 cortactin (Cell Signaling, Danvers, MA) goat anti-dynamin 2/3 (Santa Cruz Biotechnology, Santa Cruz, CA), rabbit anti-caveolin (BD Biosciences, San Jose, CA), mouse anti-tubulin and mouse anti-actin (Sigma, St. Louis, MO), rabbit anti-PhosphoTyr397 FAK (Biosource), rabbit anti-FAK (Millipore, Billerica, MA), mouse anti-cortactin (clone 4F11, Millipore, Billerica, MA), mouse anti-phospho p42/44 Erk (Santa Cruz Biotechnology, Santa Cruz, CA), rabbit anti-Erk (Cell Signaling, Danvers, MA) rabbit anti-phospho Tyr 118 paxillin (Millipore, Billerica, MA), mouse anti-paxillin (BD, Franklin Lakes, NJ), rat anti-HA (Roche, Nutley, NJ) and mouse anti-phosphotyrosine (clone 4G10, Millipore, Billerica, MA). Rabbit anti-dynamin2 was previously described [37]. Alexa546-phalloidin as well as Alexa488-phalloidin, Alexa546-cholera toxin B fragment, gelatin-Oregon green and Alexa488, 546 or 633-conjugated secondary antibodies were from Invitrogen (Invitrogen, Carlsbad, CA).

### Plasmids

pEGFP-Actin Vector was from Clontech (Palo Alto, CA). Dynamin 2aa-GFP and dynamin 2aa-ΔPRD-GFP were generously provided by Dr M. McNiven (Mayo Clinic, Rochester, MN). GFP-paxillin, VASP-RFP and cortactin-DsRed were generous gift from Dr K. Rottner (German Research Centre for Biotechnology, Braunschweig, Germany), Prof. Gertler (Koch Institute-MIT, Boston, USA) and Dr M. Kaksonen (EMBL, Heidelberg), respectively.

Dynamin 2aa-GFP and dynamin 2-ΔPRD-GFP were subcloned in the bicistronic retroviral vector pQCXIX (Clontech, Mountain View, CA). pQCXIX-Dyn2-S45N-GFP, pQCXIX-Dyn2-T65A-GFP, pQCXIX-Dyn2-K688A-GFP constructs were made using Quickchange XL site-directed mutagenesis kit (Stratagene, La Jolla, CA) using the following primers: S45N forward, CAGAGCGCCGGCAAGaAtTCGGTGCTCGAG, reverse, CTCGAGCACCGAaTtCTTGCCGGCGCTCTG; T65A for, GATCAGGAATTGTCgccCGGAGGCCTC, rev, GAGGCCTCCGggcGACAATTCCTGATC; K688A for, TGATCAACAACACAgcgGCCTTCATCCACCATG, rev, CATGGTGGATGAAGGCcgcTGTGTTGTTGATC. Mouse HA-tagged dynamin 2 was subcloned into the retroviral pBabe-puro vector and the mouse dynamin2-K562E-HA construct was obtained by using site-directed mutagenesis. Mouse HA-tagged dynamin 1 was subcloned in pQCXIX and the series of truncated dynamin mutants were obtained by PCR amplyfing starting from a BstBI internal site (corresponding to amino acid position 534) with a common forward primer, ggtcattcgaaaggggtggttgacc, and by using reverse primers matching the indicated amino acid positions: 767, TTAATTAACATGGGCGTGCTGACGGTGGTCGTGTTGATGTCG; 793, TTAATTAAGGGGCTGGACGTGGGCGATCTGC; 803, TTAATTAAGGGCACGGCGGGGGCTCGGCGCTG; 816, TTAATTAAAGGCCCAGGAGCAGGGCCCCGCGATCC; 827, TTAATTAAGGGCGCCCCCCCCAGGGCGGATCCAGC; 842, TTAATTAAGGGGCCAAAGGGGTCAGGGGAAGC. For the pTRFP or KillerRed-tagged dynamin 2aa construct, the pTRFP or KillerRed coding sequences were PCR amplified from pTagRFP-N or pKillerRed-N vectors (Evrogen, Moscow, Russia) and ligated into the pQCXIX-Dynamin 2aa plasmid (Invitrogen) to generate an in frame C-terminal fusion.

### Cell culture and infection

Dynamin 1^−/−^, dynamin 2^flox/flox^ fibroblasts were isolated from mouse embryos (embryonic day 13) and immortalized by serial passaging as described recently [1]. Yale Cancer Center Animal Genomics Shared Resource approved this study allowing to generate these cells that we used lately. Indeed, animal care and use was carried out in accordance with our institutional guidelines (Yale Cancer Center Animal Genomics Shared Resource).

Cells were grown in DMEM (containing glutamine)+10% fetal bovine serum+1% penicillin/streptomycin supplement. Cre recombinase expression was achieved via adenovirus trandusction and was purchased from the University of Iowa Gene Transfer Vector Core (Iowa City, IA). Cells were generally used for experiments between 4 and 6 days after Cre recombinase delivery. For rescue experiments, the cells were firstly transduced with exogenous dynamin constructs before addition of the CRE recombinase to remove the endogenous form of the GTPase. In most experiments, cDNAs were delivered via a retroviral transduction following packaging in Phoenix-Eco cells or Phoenix-Ampho cells (ATCC, Manassas, VA). Supernatant containing viral particles from such cells was harvested, 0.45 µm filtered and following addition of 8 µg/ml polybrene (Sigma, St Louis, MO) was used to transduce fibroblasts. The cells were serum starved for 14 h prior to experiments in order to help maximize the contribution of v-Src to overall cellular signalling.

### Degradation and invasion test

Coverslips were coated with gelatin-OregonGreen (1 mg/ml), fixed with PFA 4% (g/vol)+0.5% glutaraldehyde for 30 min at 4°C. Then, coverslips were washed with PBS and sodium borohydride (30 mg/ml), sterilized with ethanol 70% and rinsed with PBS 1×. Cells were seeded on these coverslips in culture medium and imaged for 14 h before fixation. Degradation was measured by subtracting the fluorescence intensity of the images of the gelatin layer at the beginning and end of the movies, and then dividing the total amount of pixel intensity (Intensity AU, corresponding to digested area) per the number of cells present in the field. Our invasion assay is based on the addition of a layer of BD Matrigel™ (Basement Membrane Matrix, High Concentration (HC), LDEV-Free, BD, Franklin Lakes, NJ) on a transwell permeable support (8 µm pores) (Corning Costar Corp., Corning, NY). 100 000 cells were harvested, seeded on top of the Matrigel layer in DMEM without serum and attracted towards the bottom of the transwell for 20 h by the addition of DMEM supplemented with 1% serum in the lower chamber. Cells that did not pass through the matrigel layer and through the upper part of the insert were removed. Only cells that passed through the filters were fixed and stained with crystal violet.

### Microscopy and photoinactivation

For immunofluorescence, cells were fixed with 4% paraformaldehyde in phosphate-buffered saline (PBS 1×), pH 7.4, processed as described (Ory et al., 2000), and imaged with a Zeiss Axiovert 200M equipped with a CoolSNAP HQ2, 63× (NA 1.4) Plan Apochromat and 100× (NA 1.46) Plan Apochromat objectives and a filterset to specifically detect Alexa488/GFP or Alexa546/pTRFP/KillerREd. At least 700 invadosome structures were analyzed for each condition, representative to 3–5 independent experiments.

For live imaging, cells were seeded at sub-confluent densities into serum-coated 35 mm glass bottom (thickness = 0.17 mm) dishes (Mattek, Ashland, MA) and allowed to grow for 12 to 48 hours prior to imaging. The dishes were then transferred to CO2 independent medium (Invitrogen), placed on a 37°C heated stage (Carl Zeiss Microimaging, Inc., Thornwood, NY) and imaged with the same Zeiss Axiovert 200M microscope set-up previously described. For photoinactivation experiments, we used Zeiss Axiovert 200M with a 63× (NA 1.4) Plan Apochromat to illuminate with a triple filter set 25E with individual excitation filters (405, 495, or 575, HE), beam splitter (TFT 435+510+600, HE), emission (TBP 460+530+625, HE) (Carl Zeiss microimaging GmbH, Gottingen, Germany). Cells were imaged as for live imaging before imaging was paused to allow illumination for 40 to 50 s (45 s in average) with a 100W HBO mercury lamp (100% power).

Imaging Series 7.0 (Universal Imaging, Downington, PA) was used to mount .avi movies from image stacks. Extracted images from stacks were processed with Adobe Photoshop CS2 and Adobe Illustrator CS (Adobe Systems, San Jose, CA) and Image J (http://rsb.info.nih.gov/ij/). Significance of the differences between standard deviations was analyzed in Excel with an F-test.

## Supporting Information

Figure S1 **Actin structures induced by dynamin depletion in DKO-v-Src-MEFs cells colocalize partially with clathrin light chain and present different cortactin dynamics.** A) Chlathrin light chain staining (in green) revealed that clathrin coated pits are formed around invadosomes (stained by F-actin, red, and phosphotyrosine, blue) in SKO-v-Src-MEFs. B) Dynamin depletion in DKO-v-Src-MEFs cells increased the number of arrested endocytic clathrin coated pits (green arrows) which accumulates F-actin and numerous phosphorylated proteins on tyrosine as revealed by phalloidin (red) staining and anti-phosphotyrosine (blue) in higher magnifications of the areas in the white squares. On the contrary, larger actin spots are also containing phosphorylated proteins on tyrosine but are not associated with clathrin light chains (red arrows). Scale bar = 3 µm (A, B).
(TIF)

Figure S2 **Dynamin depletion reduces migration speed without blocking it.** Extracted images from time series (h∶min) from representative observations of monolayers of SKO-v-Src-MEFs and DKO-v-Src-MEFs that migrate in response to a wound. Some cells were tracked overtime and the colored lines represent the total distance realized during the test. Scale bar = 20 µm.
(TIF)

Figure S3 **The effect of dynamin depletion on invadopodia is poorly correlated with signaling events downstream of adhesion structures.** A) In MEF-SKO-v-Src, the specific effects of the depletion of dynamin on signaling molecules involved in invadosome régulation was analyzed by western-blot: membranes blotted with phosphospecific antibodies were then stripped and reprobed to show the total amount of each protein. With the exception of phospho-Erk (red square), dynamin depletion does not alter the level of phosphorylation of Fak, cortactin and Src. B) Quantification and normalization of the number of invadosome rings in MEF-SKO-v-Src and MEF-DKO-v-Src treated with increasing concentrations of the Erk inhibitor UO126; Erk inhibition decreases invadosome formation in a dose dependent manner in MEF-SKO-v-Src. In contrast, there is no rescue in MEF-DKO-v-Src despite gradual inhibition of Erk.
(TIF)

Figure S4 **Specific dynamin photoinactivation led to the rapid invadosome desorganization.** Distribution of the percentage of cells where invadosome structures is disorganized×min after light irradiation. 16 to 56 cells per conditions were monitored. Scale bar = 2 µm (A, E), 4 µm (B, C, D).
(TIF)

Movie S1 **Live imaging (0,016 Hz) of invadosome ring visualized by dyn2-pTRFP associated with a degradation activity of a layer of gelatinOr.Green.**
(AVI)

Movie S2 **Live imaging (0,016 Hz) of invadosome ring visualized by GFP-actin and dyn2-pTRFP.**
(AVI)

Movie S3 **Live imaging (0,066 Hz) of SKO-v-Src-MEFs (left) and DKO-v-Src-MEFs expressing GFP-actin.**
(AVI)

Movie S4 **Live imaging (0,066 Hz) of invadosome ring present in a DKO-v-Src-MEFs epxressing paxillin-GFP and Dyn2-KR, followed by 45 s of red light irradiation to photo-inactivate Dyn2-KR.**
(AVI)

Movie S5 **Live imaging (0,066 Hz) of invadosome ring present in a SKO-v-Src-MEFs epxressing paxillin-GFP and KillerRed™ alone, followed by 45 s of red light irradiation to photo-inactivate KillerRed™.**
(AVI)

Movie S6 **Live imaging (0,066 Hz) of invadosome ring present in a DKO-v-Src-MEFs epxressing paxillin-GFP and Dyn2-pTRFP, followed by 45 s of red light irradiation of Dyn2-pTRFP.**
(AVI)

Movie S7 **Live imaging (0,066 Hz) of invadosome ring present in a SKO-v-Src-MEFs epxressing paxillin-GFP and Dyn2-KR, followed by 45 s of red light irradiation to photo-inactivate Dyn2-KR.**
(AVI)

Movie S8 **Live imaging (0,066 Hz) of invadosome ring present in a DKO-v-Src-MEFs epxressing Dyn2-GFP and Dyn2-KR, followed by 45 s of red light irradiation to photo-inactivate Dyn2-KR.**
(AVI)

Movie S9 **Live imaging (0,033 Hz) of invadosome ring visualized by GFP-paxillin and dyn2-pTRFP.**
(AVI)

Movie S10 **Live imaging (0,033 Hz) of invadosome ring visualized by GFP-cortactin and dyn2-pTRFP.**
(AVI)
