# Supplementary figures and images for: Essential Function of Dynamin in the Invasive Properties and Actin Architecture of v-Src Induced Podosomes/Invadosomes
Source: PLoS One. 2013 Dec 9;8(12):e77956. doi: 10.1371/journal.pone.0077956 (PMC3857171; doi:10.1371/journal.pone.0077956)

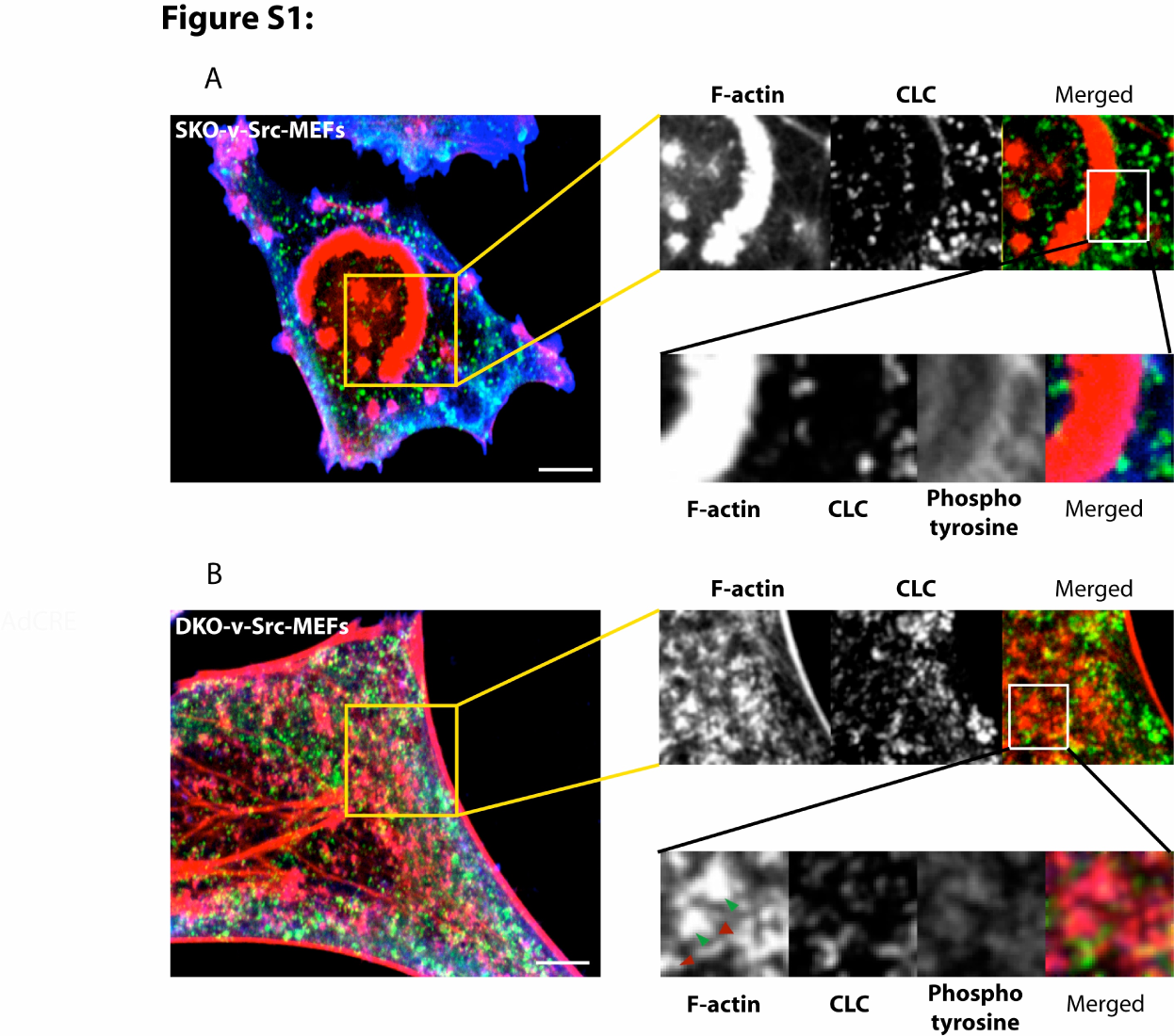

Supplement: Figure S1 — Actin structures induced by dynamin depletion in DKO-v-Src-MEFs cells colocalize partially with clathrin light chain and present different cortactin dynamics. A) Chlathrin light chain staining (in green) revealed that clathrin coated pits are formed around invadosomes (stained by F-actin, red, and phosphotyrosine, blue) in SKO-v-Src-MEFs. B) Dynamin depletion in DKO-v-Src-MEFs cells increased the number of arrested endocytic clathrin coated pits (green arrows) which accumulates F-actin and numerous phosphorylated proteins on tyrosine as revealed by phalloidin (red) staining and anti-phosphotyrosine (blue) in higher magnifications of the areas in the white squares. On the contrary, larger actin spots are also containing phosphorylated proteins on tyrosine but are not associated with clathrin light chains (red arrows). Scale bar = 3 µm (A, B). (TIF) [file pone.0077956.s001.tif]

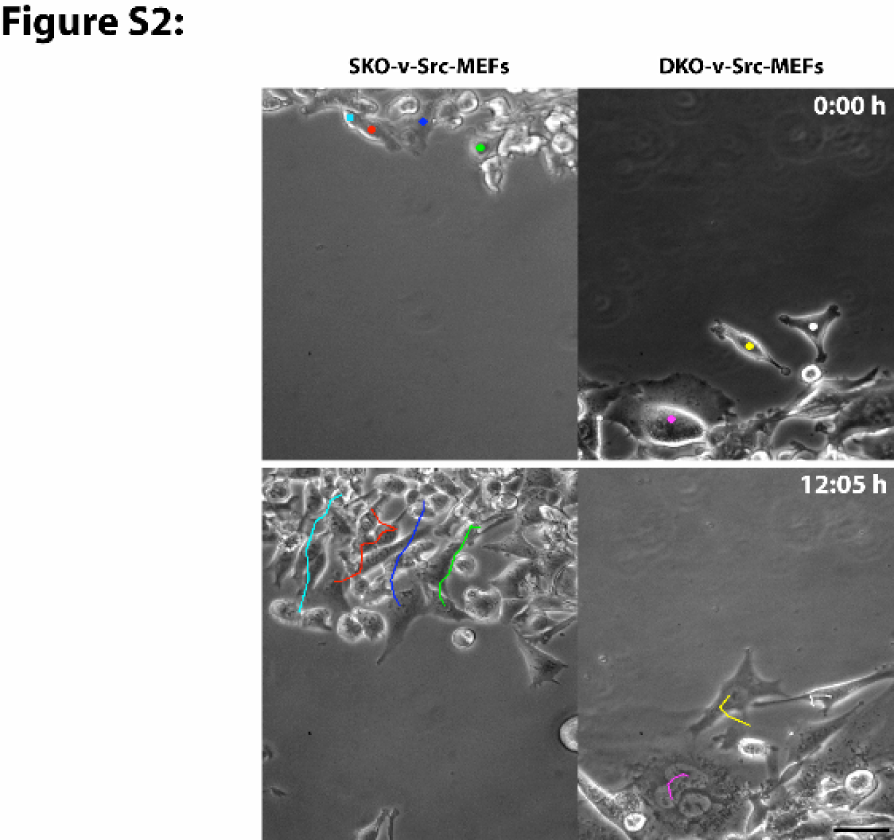

Supplement: Figure S2 — Dynamin depletion reduces migration speed without blocking it. Extracted images from time series (h∶min) from representative observations of monolayers of SKO-v-Src-MEFs and DKO-v-Src-MEFs that migrate in response to a wound. Some cells were tracked overtime and the colored lines represent the total distance realized during the test. Scale bar = 20 µm. (TIF) [file pone.0077956.s002.tif]

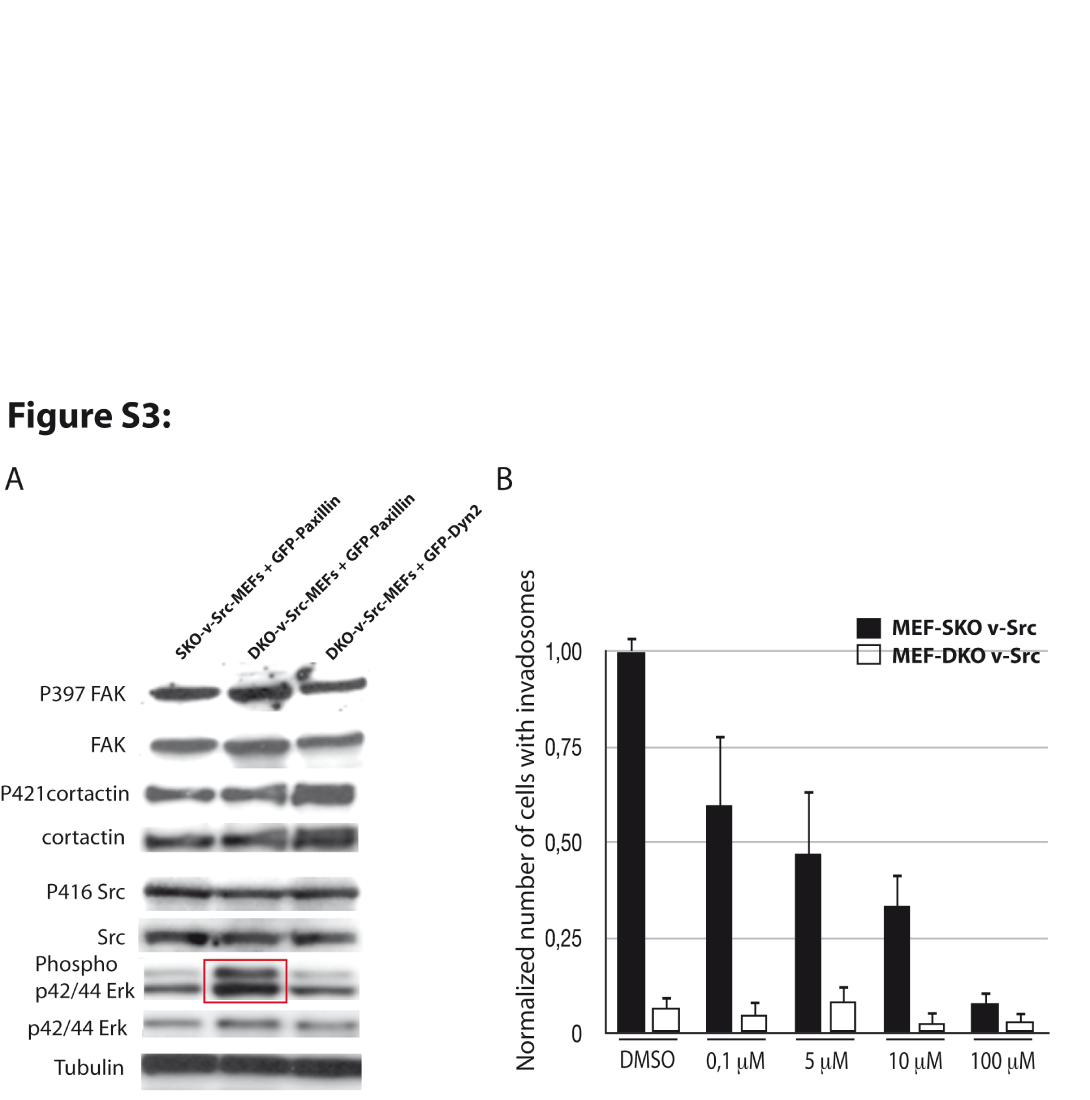

Supplement: Figure S3 — The effect of dynamin depletion on invadopodia is poorly correlated with signaling events downstream of adhesion structures. A) In MEF-SKO-v-Src, the specific effects of the depletion of dynamin on signaling molecules involved in invadosome régulation was analyzed by western-blot: membranes blotted with phosphospecific antibodies were then stripped and reprobed to show the total amount of each protein. With the exception of phospho-Erk (red square), dynamin depletion does not alter the level of phosphorylation of Fak, cortactin and Src. B) Quantification and normalization of the number of invadosome rings in MEF-SKO-v-Src and MEF-DKO-v-Src treated with increasing concentrations of the Erk inhibitor UO126; Erk inhibition decreases invadosome formation in a dose dependent manner in MEF-SKO-v-Src. In contrast, there is no rescue in MEF-DKO-v-Src despite gradual inhibition of Erk. (TIF) [file pone.0077956.s003.tif]

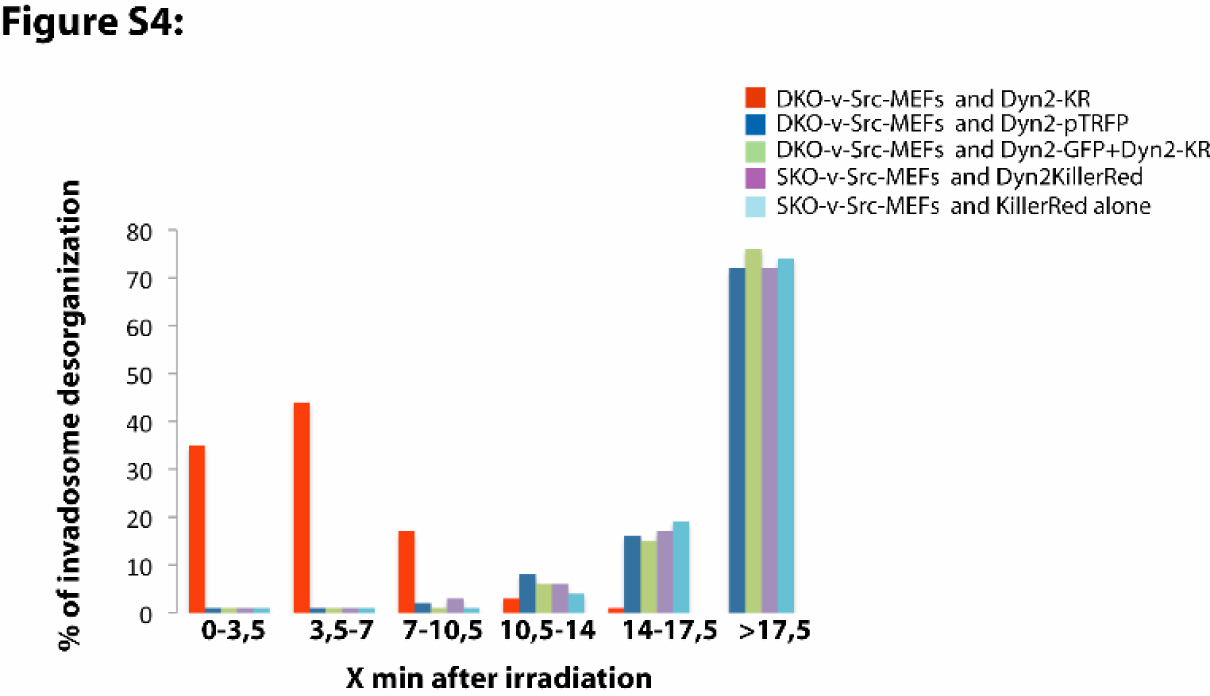

Supplement: Figure S4 — Specific dynamin photoinactivation led to the rapid invadosome desorganization. Distribution of the percentage of cells where invadosome structures is disorganized×min after light irradiation. 16 to 56 cells per conditions were monitored. Scale bar = 2 µm (A, E), 4 µm (B, C, D). (TIF) [file pone.0077956.s004.tif]
